# *ChnagG* Plays the Role of 5‐Salicylate Hydroxylase in the Gentisic Acid Pathway of Salicylic Acid Metabolism in *Cochliobolus heterostrophus*

**DOI:** 10.1111/mpp.70090

**Published:** 2025-06-02

**Authors:** Yadi Xu, He Wei, Haixiao Li, Fanli Zeng, Ning Liu, Zhiyan Cao, Jingao Dong

**Affiliations:** ^1^ State Key Laboratory of North China Crop Improvement and Regulation/Hebei Key Laboratory of Plant Physiology and Molecular Pathology Baoding China; ^2^ College of Life Sciences Hebei Agricultural University Baoding China; ^3^ College of Plant Protection Hebei Agricultural University Baoding China

**Keywords:** *Cochliobolus heterostrophus*, gentisic acid, maize ( *Zea mays* ), pathogenicity, salicylate hydroxylase, salicylic acid

## Abstract

Salicylic acid (SA) plays a crucial role in the defence strategies of plants against fungal pathogens. To circumvent plant immunity, pathogens use metabolic enzymes such as salicylate hydroxylase to degrade SA, thereby facilitating successful pathogenicity after infection. This phenomenon has not been previously reported in *Cochliobolus heterostrophus*. Our study demonstrates that high concentrations of SA can inhibit both growth and spore germination; however, at concentrations below 1 mM, SA does not significantly impact the growth and spore germination of *C*. *heterostrophus*, which is capable of metabolising exogenously supplied SA. Transcriptome and LC–MS analyses indicated that *C*. *heterostrophus* metabolises exogenous SA via the gentisic acid (GA) pathway, involving genes such as 5‐salicylate hydroxylase (*ChnagG*). Prokaryotic expression of *ChnagG* confirmed its ability to convert SA into GA. Additionally, we created *ChnagG* gene deletion and complementation mutants, revealing that *ChnagG* influences melanin synthesis and the pathogenicity of *C*. *heterostrophus*. Analysis of the SA signalling pathway in plants during fungal infection indicated that the *ChnagG* knockout mutant did not alter the synthesis of SA in its host maize; however, it led to the upregulation of the downstream signalling pathway *ZmPR1* gene compared to the wild type. These findings suggest that *C*. *heterostrophus* obstructs the immune signalling pathway of maize through SA metabolism, thereby enhancing its infection and pathogenicity. This study lays the groundwork for further elucidating the mechanisms underlying the interaction between maize and *C*. *heterostrophus*.

## Graphical abstract

The *ChnagG* gene in *Cochliobolus heterostrophus* encodes 5‐salicylate hydroxylase, converting salicylic acid (SA) to gentisic acid, enhancing pathogenicity and suppressing plant SA signalling‐mediated defences during infection.

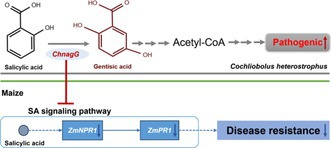

## 1 Introduction

Plants defend themselves against pathogenic microorganisms by activating various defence mechanisms, including the synthesis of their own hormones. Salicylic acid (SA), a well‐known plant defence hormone, can directly or indirectly mediate plant disease resistance (Peng et al. 2021). This includes the activation of various antioxidant enzymes (Iqbal et al. 2021), the promotion of antimicrobial compounds (Ali et al. 2018; Wang et al. 2022a; Vañó et al. 2023), the triggering of pathogenesis‐related (PR) proteins (Dos Santos and Franco 2023) and even the induction of programmed cell death (Iqbal et al. 2021), all of which enhance the plant's tolerance to biotic stress caused by pathogens, fungi and pests (Song et al. 2023). Conversely, pathogenic microorganisms employ various strategies to evade plant defence mechanisms (Wang et al. 2022b), such as obstructing host immune signal transduction, reprogramming host immune responses, evading immune recognition and adapting to changes in immune microenvironments (Chen et al. 2021). Throughout this interaction, plants and pathogens both continuously evolve and adapt to establish a relatively stable balance (Delaux and Schornack 2021). To counteract the direct or indirect inhibition of pathogenic infection by SA, pathogenic fungi impede SA‐mediated defences by converting SA into inactive derivatives (Qi et al. 2018a).

Microorganisms metabolise SA through various enzymatic pathways. Existing literature indicates that bacteria use six distinct pathways for SA metabolism, including the catechol pathway, whereas reports concerning fungi are less prevalent. In filamentous fungi, it has been confirmed that SA can be metabolised into catechol and salicylaldehyde, while in yeast, SA is converted into phenol (Lubbers et al. 2019). Among them, salicylic acid hydroxylase (E1.14.13.1), encoded by the gene *FgNahG* in *Fusarium graminearum*, facilitates the conversion of SA to catechol, thereby reducing SA accumulation and aiding the pathogen in overcoming host defences (Qi et al. 2019b). The catechol pathway has also been validated in other fungal species (Anderson and Dagley 1980; Milstein et al. 1983; Kuswandi and Roberts 1992; Kirimura et al. 2010; Penn and Daniel 2013; Martins et al. 2015). Alternatively, SA can first undergo hydroxylation to form 2,3‐dihydroxybenzoic acid, which is subsequently metabolised to catechol. For example, the Sdc enzyme from *Trichoderma moniliiforme* and the 2,3‐dihydroxybenzoic acid decarboxylase from *Aspergillus niger* have been shown to catalyse the decarboxylation of 2,3‐dihydroxybenzoic acid ester to catechol (Santha et al. 1995; Kirimura et al. 2010); the production of salicylaldehyde has been documented in *Neurospora crassa*; however, the specific enzymes responsible for these conversions remain unidentified (Bachman et al. 1960). Furthermore, the *BsdBCD* operon from *Bacillus subtilis* and *Sdc* from *Trichoderma moniliiforme* facilitate the decarboxylation of SA to phenol, yet there has been a lack of relevant research on this pathway in filamentous fungi (Lupa et al. 2008; Kirimura et al. 2010). The downstream metabolites of SA, including gentisic acid (GA), salicyl‐AMP and 2‐oxidoethyl‐3,5‐dienedioic acid, have only been identified in bacteria (Grund et al. 1990; Fuenmayor et al. 1998; Hintner et al. 2001; Zhou et al. 2001; Ishiyama et al. 2004; Matera et al. 2008). In *Ralstonia solanacearum* , SA is converted by the key genes *nagG* and *nagH* to produce GA, with subsequent metabolic products predicted to include maleylpyruvic acid and pyruvic acid, neither of which are considered active compounds for plant defence signal transduction (Fuenmayor et al. 1998; Zhou et al. 2001). However, the enzymes responsible for the conversion of SA to GA have not yet been identified in fungi. Clarifying the metabolic pathways of SA in plant pathogens is essential for understanding the SA signalling immune pathways that pathogenic fungi use to evade plant defences during interactions.

Despite some progress in elucidating the interplay between plant‐pathogenic microorganisms and SA defence pathways, there have been no reports specifically addressing this in *Cochliobolus heterostrophus*. This pathogen is responsible for southern corn leaf blight (SCLB), one of the major leaf diseases affecting maize, which occurs in nearly all maize‐growing regions worldwide and significantly impacts crop yield and quality. *C*. *heterostrophus* primarily attaches to the host surface via conidia, accumulates melanin to form expanded infection structures, and subsequently penetrates the leaf cuticle. Following this initial infection, it spreads to other parts of the plant through hyphae, resulting in the formation of characteristic lesions (Shimizu et al. 1997; Eliahu et al. 2007; Izumitsu et al. 2009; Lev et al. 2009). Furthermore, *C*. *heterostrophus* can synthesise mycotoxins to facilitate its infection, with extensive research conducted on the synthesis mechanisms of T‐toxins (Igbaria et al. 2008; Inderbitzin et al. 2010; Oide et al. 2010; Wu et al. 2012). Transcriptomic analyses indicate that during infection, various genes associated with peroxisomes, energy metabolism, amino acid degradation and oxidative phosphorylation play crucial roles in the virulence of *C*. *heterostrophus* (Yu et al. 2024). However, there is a paucity of reports detailing how *C*. *heterostrophus* overcomes host defences.

To analyse the key genes associated with the SA metabolic pathways in *C*. *heterostrophus*, this study investigated the primary differentially expressed genes (DEGs) and related metabolites in response to exogenous SA treatment, thereby providing a preliminary overview of the SA metabolic pathway. Furthermore, we performed a functional analysis on the key gene *ChnagG*. Our findings elucidate the metabolic pathways of SA in *C*. *heterostrophus*, contributing a theoretical foundation for exploring the interaction mechanisms between *C*. *heterostrophus* and maize, and offering innovative insights for managing maize disease resistance.

## 2 Results

### 2.1 *C*. *heterostrophus* Can Utilise Exogenous SA

**FIGURE 1:**
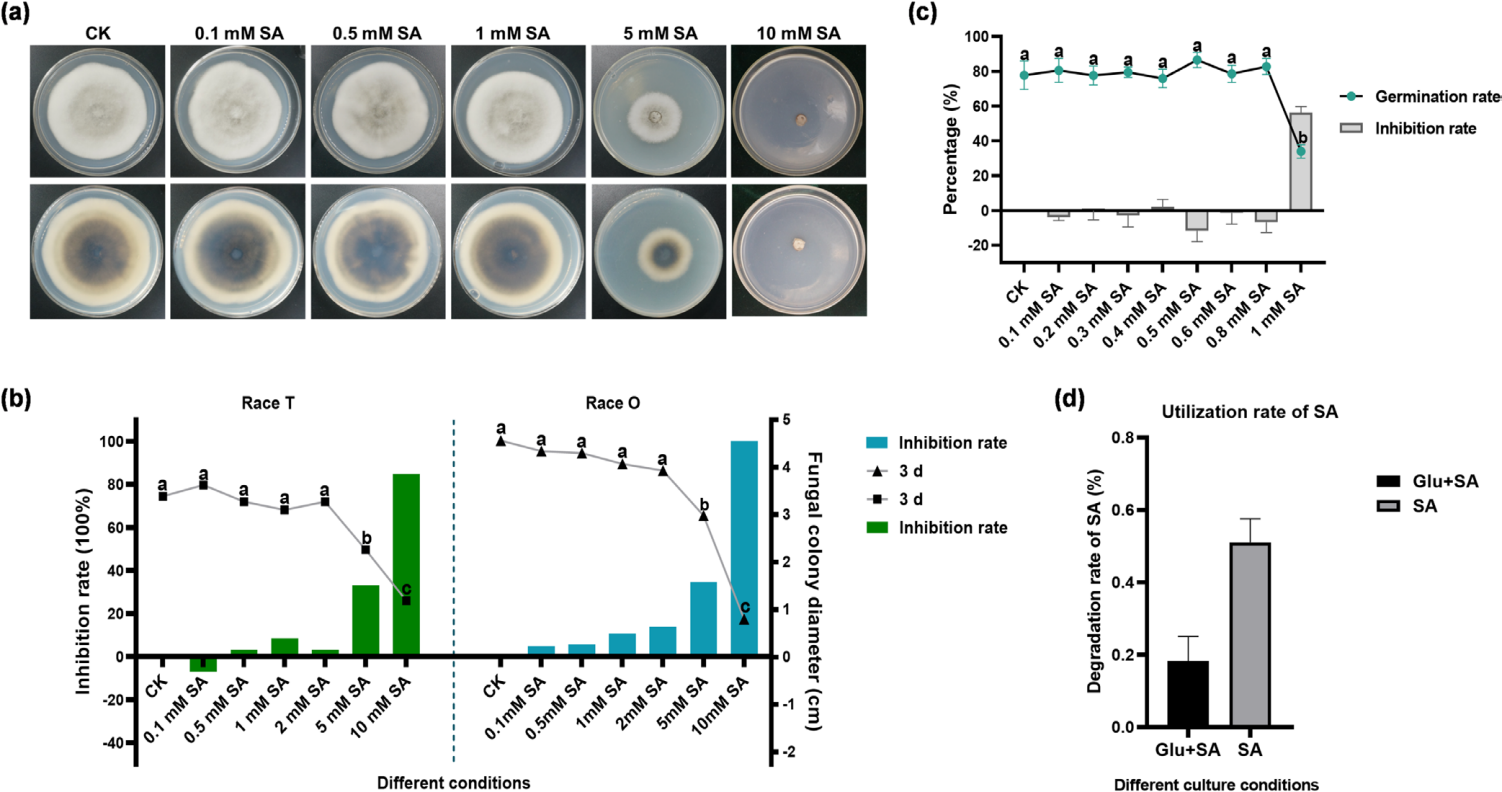
The effect of salicylic acid (SA) on the growth and development of *Cochliobolus heterostrophus*. All experiments were conducted with three independent biological replicates, and the results from these experiments are presented as means and SE (bar). Independent tests for means equality indicated that the differences between the control group and the treatment group were highly significant (*p* < 0.01). (a) The growth status of *C*. *heterostrophus* (race T) mycelium on complete medium (CM) with added SA. The SA concentrations tested were 0 (only methanol, CK), 0.1, 0.5, 1, 5 and 10 mM. (b) The mycelial growth rates of *C*. *heterostrophus* (race T and race O) on CM with varying concentrations of SA; the diameters of the fungal colonies were measured continuously over 7 days, and significance difference analysis was performed on the data collected at the 3‐day mark, corresponding to the phenotypes shown in (a). The data are presented as the mean ± SD based on triplicate measurements from a representative experiment. Significant differences between groups (*p* < 0.05) were analysed by one‐way ANOVA, groups labelled with the same lowercase letter (a, b and c) are not statistically different. Similar results were obtained in the next independent experiments. (c) The spore germination rate of *C*. *heterostrophus* (race T) mycelium on CM with different concentrations of SA. (d) High‐performance liquid chromatography (HPLC) detection of SA content in the culture medium. ‘Glu + SA’ for the presence of both glucose (10 g/L) and SA, while ‘SA’ indicates that only SA is present in culture medium without glucose.

When SA was directly administered, low concentrations (0.1–1 mM) did not significantly inhibit the growth of race T mycelia. However, at a concentration of 2 mM, the presence of SA resulted in a significant reduction in mycelial growth, and at 10 mM, the fungus completely ceased growth (Figure 1a,b). Similar patterns were observed for race O (Figure 1b). On water agar plates, 1 mM SA significantly inhibited the germination of conidia compared to the control (Figure 1c), whereas concentrations below 1 mM had no discernible effect. We eliminated the possibility that the culture medium's pH, within the range of 0.1 mM to 1 mM SA, influenced the growth of *C*. *heterostrophus* (Figure S1a,b). These findings suggest that at lower concentrations, SA does not affect the growth and spore germination of *C*. *heterostrophus*; conversely, at higher concentrations, it significantly inhibits both spore germination and mycelial growth.

To assess the metabolism of SA by *C*. *heterostrophus*, high‐performance liquid chromatography (HPLC) was used to quantify the residual SA in the culture medium after a 3‐day incubation with added exogenous SA. In comparison to the uninoculated medium, the degradation rate of 0.5 mM SA in glucose‐containing medium after 3 days was 15.3%. Conversely, in the absence of glucose, the degradation rate increased significantly to 51.0% over the same period (Figure 1d). These results indicate that *C*. *heterostrophus* can directly utilise SA.

### 2.2 Transcriptome Analysis of SA's Effect on Gene Expression in *C*. *heterostrophus*

**FIGURE 2:**
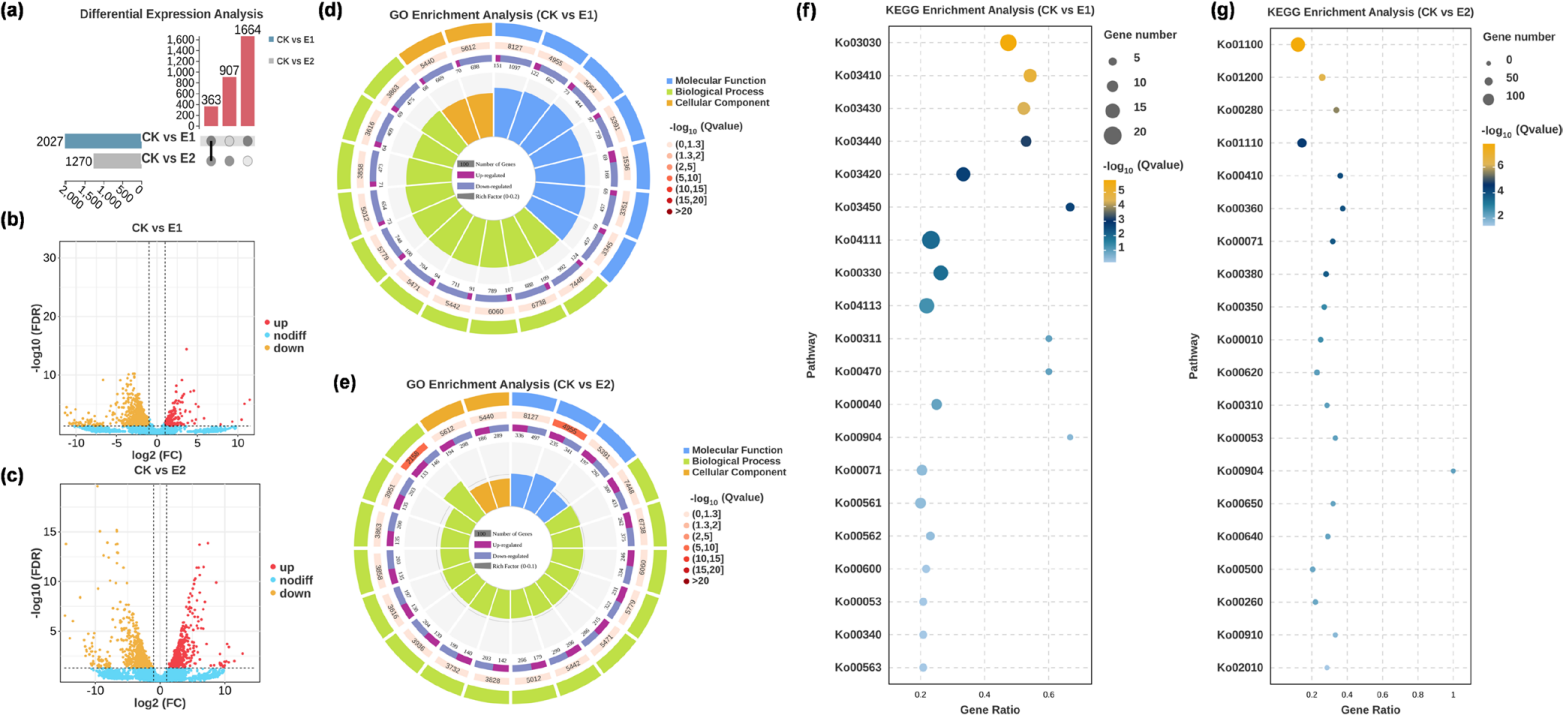
Analysis of transcriptomic data for *Cochliobolus heterostrophus* under various treatments. The cultivation conditions modified the carbon source composition of the medium as follows: Glucose + methanol (CK), glucose +0.5 mM salicylic acid (SA) (Experiment 1, E1), and 0.5 mM SA (Experiment 2, E2). (a) Statistical data on differential gene expression across different treatments. (b, c) Differential gene status between groups. The scatter points represent differentially expressed upregulated and downregulated genes filtered by the specified threshold, with varying colours indicating different differential trends. (b) The differential genes for CK versus E1; (c) the differential genes for CK versus E2. (d, e) GO analysis of the transcriptomic data. Molecular functions (MF), biological processes (BP) and cellular components (CC) categorised in blue, green and yellow, respectively. The first circle of the enrichment diagram distinguishes different ontologies by colour; the second circle indicates the number of genes enriched in the GO terms, and the third circle displays the number of differential genes concentrated in these terms, with different colours representing trends in gene changes. The innermost circle represents the gene ratio (gene ratio = number of differential genes enriched in the GO term/number of differential genes in the background that are enriched in the GO term), with colours corresponding to those in the first circle. (f, g) KEGG analysis of transcriptomic data. The size of the bubbles reflects the number of differential genes enriched in each pathway, while the colour of the bubbles indicates the significance of enrichment, with larger values denoting greater significance. Furthermore, the gene ratio is calculated as the number of differential genes enriched in the pathway divided by the total number of background genes associated with that pathway.

To clarify the effect of SA on gene expression in *C*. *heterostrophus*, high‐throughput RNA sequencing was conducted on samples cultivated under various culture conditions: glucose combined with methanol (negative control, CK), glucose supplemented with 0.5 mM SA (E1) and 0.5 mM SA alone (E2). The results indicated that the addition of SA as a carbon source alongside glucose (CK vs. E1) resulted in the identification of 2027 DEGs, of which 224 genes were upregulated and 1803 genes were downregulated (Figure 2a). To elucidate the biological functions of the selected DEGs, we performed Gene Ontology (GO) and KEGG pathway analyses. The upregulated genes were significantly enriched in pathways associated with essential cellular activities, including molecular function (GO:0003674), biological process (GO:0008150) and cellular process (GO:0009987). Additionally, these genes influenced the expression of genes linked to metabolic pathways, such as those involved in catalytic activity (GO:0003824) and metabolic processes (GO:0008152) (Figure 2b). The KEGG pathway analysis of significantly different genes revealed that SA directly impacts fundamental life activities within the fungus (e.g., Ko03030, Ko03410 and Ko03430) (Figure 2c).

In contrast, when the medium contained only SA, a comparison with the control group (CK vs. E2) revealed a total of 1270 DEGs, comprising 498 upregulated and 772 downregulated genes (Figure 2a). GO term enrichment analysis indicated that the upregulated DEGs were predominantly enriched in cellular anatomical entity (GO:0110165), nitrogen compound metabolic process (GO:0006807) and organic cyclic compound metabolic processes (GO:1901360), highlighting the regulatory effect of SA on fungal cellular structure and material metabolism (Figure 2d). Furthermore, KEGG pathway analysis showed that the DEGs were enriched in metabolic pathways (Ko01100), carbon metabolism (Ko01200), valine, leucine and isoleucine degradation (Ko00280), biosynthesis of secondary metabolites (Ko01110) and β‐alanine metabolism (Ko00410), indicating a shift in the fungal metabolic mode following the substitution of glucose with SA as a carbon source (Figure 2e).

Gene Ontology (GO) enrichment analysis comparing both treatments to the control group revealed that the upregulated genes were primarily associated with catalytic activity (GO:0003824), cellular processes (GO:0009987) and metabolic processes (GO:0008152). This suggests that the addition of SA has an impact on the metabolism of *C*. *heterostrophus* (Figure S2).

### 2.3 Prediction of the GA Pathway for SA Metabolism in *C*. *heterostrophus*

**FIGURE 3:**
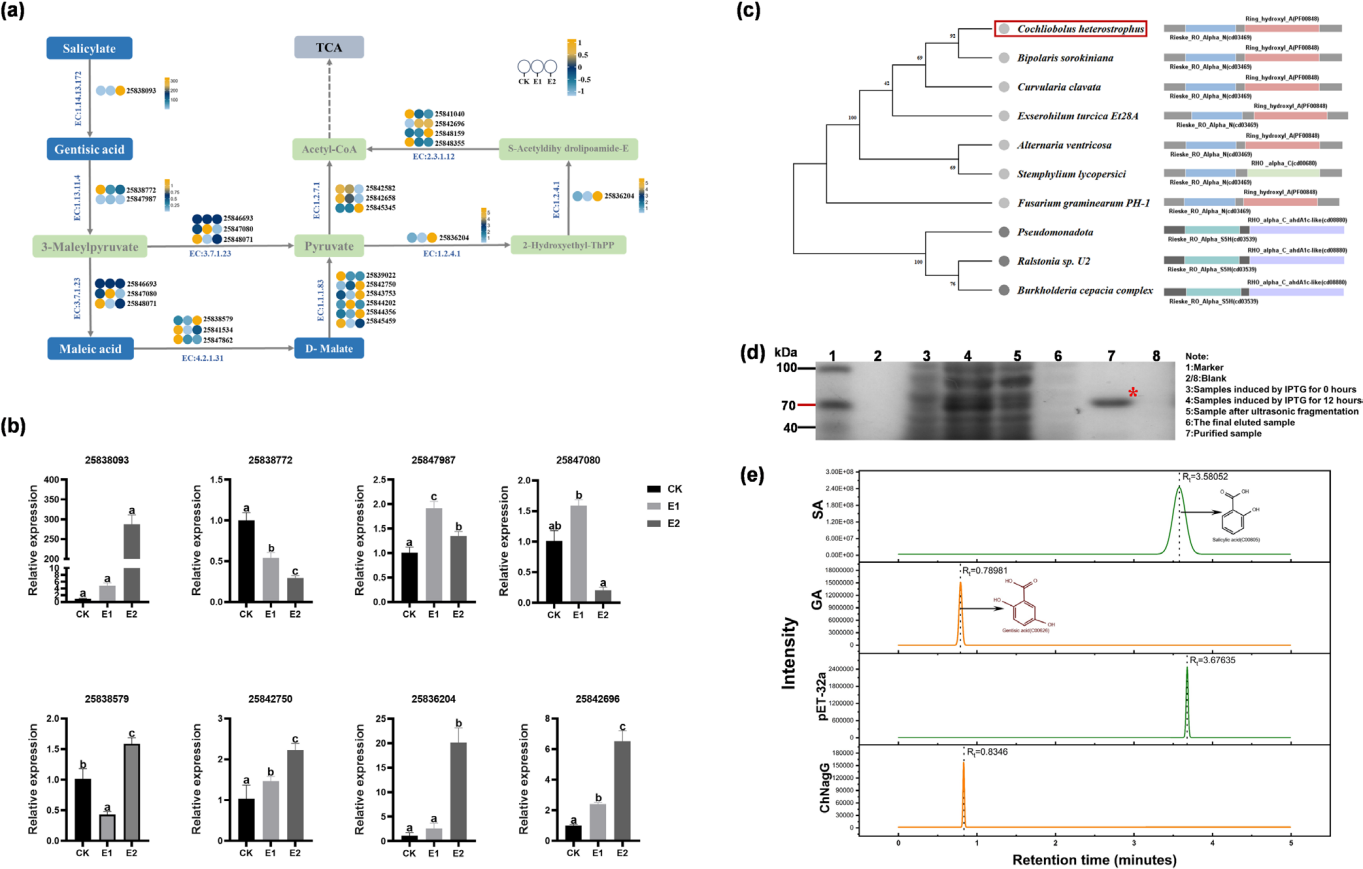
Prediction of the salicylate metabolism pathway in *Cochliobolus heterostrophus*. (a) The predicted pathway for salicylic acid (SA) metabolism in *C*. *heterostrophus* is illustrated, with substances detected by high‐performance liquid chromatography (HPLC) highlighted in blue boxes. The enzymatic reactions and corresponding gene relative expression heat maps are indicated between the substances, using data from transcriptomic analysis. (b) The relative expression level of the target gene was detected. The reverse transcription‐quantitative PCR validation of the relative expression levels of target genes is presented. The data are presented as the mean ± SD based on triplicate measurements from a representative experiment. Significant differences between groups (*p* < 0.05) were analysed by one‐way ANOVA, groups labelled with the same lowercase letter (a, b and c) are not statistically different. (c) A phylogenetic tree and domain analysis of homologous genes is provided, with light grey representing fungal protein sequences and dark grey representing bacterial protein sequences. The sequences are listed in the following order from top to bottom: XP_014077270.1 (*Cochliobolus heterostrophus*), KAF5846348.1 (*Bipolaris sorokiniana*), USP81990.1 (*Curvularia clavata*), XP_008025777.1 (*Exserohilum turcica* Et28A), XP_049202630.1 (*Alternaria ventricosa*), KNG52537.1 (*Stemphylium lycopersici*), XP_011322893.1 (*Fusarium graminearum* PH‐1), WP_026437495.1 (*Pseudomonadota*), AAD12607.1 (*Ralstonia* sp. U2) and WP_048805403.1 ( *Burkholderia cepacia* complex). (d) SDS‐PAGE analysis of the purified recombinant protein ChNagG is shown, with the target protein indicated by a red asterisk. (e) HPLC chromatograms demonstrating analytical profiles in the following order (top to bottom): Salicylic acid standard, gentisic acid standard, negative control (*Escheichia coli* lysate containing pET‐32a empty vector) and recombinant protein ChNagG reaction system.

To investigate the SA metabolic pathway in *C*. *heterostrophus*, we initially performed liquid chromatography‐mass spectrometry (LC–MS) analysis on metabolites from *C*. *heterostrophus* cultures that were supplemented with exogenous SA for 3 days. In total, we identified 1594 compounds in positive ion mode and 1047 compounds in negative ion mode (Tables [Link], [Link] and [Link], [Link]). Among the compounds detected that are pertinent to SA metabolism were SA, GA, maleic acid and d‐malate. According to KEGG pathway annotations, these metabolites are associated with naphthalene degradation (map00626), tyrosine metabolism (map00350), butanoate metabolism (map00650) and the citrate cycle (tricarboxylic acid [TCA] cycle, map00020). Further investigation using transcriptomic data assessed the expression of genes associated with the aforementioned pathways. Based on the metabolites and DEGs, the potential SA degradation pathway was predicted in *C*. *heterostrophus* (Figure 3a). SA is first converted to GA by salicylate hydroxylase (EC: 1.14.13.172), followed by the formation of maleic acid from the intermediate 3‐maleylpyruvate via maleylpyruvate hydrolase (EC: 3.7.1.23). Subsequently, d‐malate is produced by the action of d‐malate hydro‐lyase (EC: 4.2.1.31). Through additional enzymatic reactions, acetyl‐CoA is generated and enters the TCA cycle, supplying energy for *C*. *heterostrophus*.

The results indicated that following the addition of SA (E1) to the culture medium, there were significant upregulations in the expression of candidate genes for gentisate 1,2‐dioxygenase (EC: 1.13.11.4) with candidate gene 25847987, maleylpyruvate hydrolase (EC: 3.7.1.23) with candidate gene 25847080, d‐malate dehydrogenase (EC: 1.1.1.83) with candidate genes 25842750 and 25844202 and pyruvate dehydrogenase 2 (EC: 2.3.1.12) with candidate gene 25842696. Conversely, when only SA (E2) was added to the culture medium, the expression of the key gene for 5‐salicylic acid hydroxylase (EC: 1.14.13.172) with key gene 25838093, the candidate gene for maleate hydratase (EC: 4.2.1.31) with candidate gene 25838579, d‐malate dehydrogenase (EC: 1.1.1.83) with candidate genes 25842750, 25843753 and 25844356, the key gene for pyruvate dehydrogenase 1 (EC: 1.2.4.1) with gene 25836204, pyruvate dehydrogenase 2 (EC: 2.3.1.12) with candidate genes 25842696 and 25848159 and pyruvate kinase (EC: 1.2.7.1) with candidate gene 25845345 were all significantly upregulated (Figure 3a). Using reverse transcription‐quantitative PCR (RT‐qPCR), we validated the expression differences of eight genes (25838093, 25838772, 25847987, 25847080, 25838579, 25842750, 25836204 and 25842696), and the results were consistent with the trends observed in the transcriptomic data (Figure 3b).

In subsequent studies, we focused on gene 25838093 (COCC4DRAFT_143229) within the aforementioned pathway. RT‐qPCR results indicated that the expression of this gene was significantly upregulated following the exogenous addition of SA to the culture medium, showing an upregulation of approximately 288 times compared to the control when SA was added without glucose, thereby highlighting its role in SA metabolism. We queried the homologous proteins encoded by this gene using BLAST and constructed a phylogenetic tree, which revealed the presence of homologous proteins in fungi such as *Bipolaris sorokiniana*, *Curvularia clavata* and *Exserohilum turcica*. These protein sequences contained a conserved domain, Rieske_RO_Alpha_N (cd03469); however, the functions of these homologous proteins remain unexplored (Figure 3c). Further analysis revealed the presence of the *nagG* gene in *Pseudomonadota*, *Ralstonia* sp. U2, and the *Burkholderia cepacia* complex. This gene encodes 5‐salicylic acid hydroxylase, which catalyses the conversion of SA to GA. The similarity of the NagG protein to the protein encoded by gene 25838093 of *C*. *heterostrophus* ranged from 15.1 to 15.3. Although we did not identify other S5H subunits in the *C*. *heterostrophus* genome, the Rieske Ro_Alpha_N (cd03469) domain of fungal proteins and the Rieske Ro_Alpha_S5H (cd03539) domain present in the NagG protein belong to the same protein domain family, known as Rieske (pfam00355) (accession: cl00938, PSSM Id: 445190). The Rieske non‐heme iron oxygenase (RO) family comprises a large class of aromatic ring‐hydroxylating dioxygenases predominantly found in microorganisms. Notably, the ring hydroxylating α subunit of *C*. *heterostrophus* (PF00848) and the bacterial RHO_alpha_C_ahdA1c‐like (cd08880) also belong to the START/RHO_alpha_C/PITP/Bet_v1/CoxG/CalC (SRPBCC) ligand‐binding domain superfamily (accession: cl14643, PSSM Id: 472699). This family encompasses the C‐terminal catalytic domain of the α‐oxygenase subunit of the Rieske‐type non‐heme iron aromatic cyclohydroxylated oxygenase (RHOs_alpha_C), wherein the active site contains a non‐heme ferrous ion coordinated by three ligands. These enzymes enable microorganisms to tolerate and even exclusively utilise aromatic compounds for growth. Thus, we hypothesise that the two genes may have similar functions (Figure 3c), with gene 25838093 designated as *ChnagG*.

**FIGURE 4:**
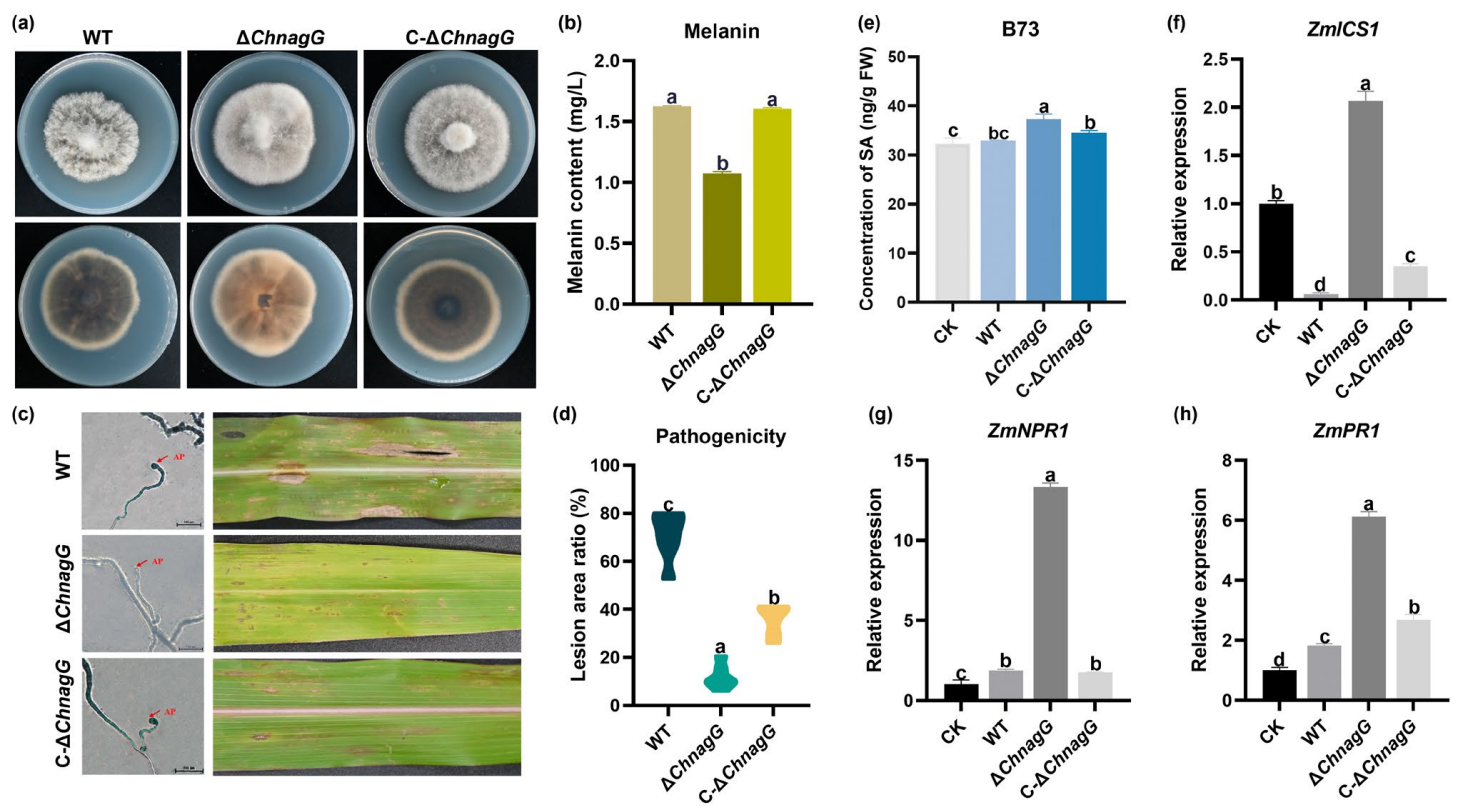
Functional identification of the salicylic acid hydroxylase gene *ChnagG*. (a) Colony phenotype of strains. WT, wild type. (b) Melanin content of strains. The data are presented as the mean ± SD based on triplicate measurements from a representative experiment. Significant differences between groups (*p* < 0.05) were analysed by one‐way ANOVA, groups labelled with the same lowercase letter (a, b and c) are not statistically different. Similar results were observed in follow‐up independent experiments. (c) Appressorium (AP) formation and pathogenicity assay. (d) Statistical results of lesion area, calculated as total lesion area divided by leaf area, correspond to Figure 4c. (e) Salicylic acid (SA) content of maize leaves (B73) at 72 h post‐inoculation with different strains. (f) Relative expression levels of SA synthesis gene *ZmICS1* in maize leaves inoculated with water, WT, Δ*ChnagG*, C‐Δ*ChnagG*. (g) Relative expression levels of SA receptor gene *ZmNPR1* in maize leaves inoculated with water, WT, Δ*ChnagG*, C‐Δ*ChnagG*. (h) Relative expression levels of *ZmPR1* in maize leaves inoculated with water, WT, Δ*ChnagG*, C‐Δ*ChnagG*.

To assess the catalytic ability of the *ChnagG‐*encoded protein on SA, we heterologously expressed the recombinant ChNagG protein in *Escherichia coli* (Figure 3d). Following 1 h incubation of the purified ChNagG protein with SA, GA was analysed in the system, while incubation with the cell lysate of *E. coli* transformed with the pET‐32a vector was a negative control; HPLC analysis detected only SA in the system, with no GA detected. This confirms that the recombinant ChNagG catalysed the hydroxylation of SA to GA (Figure 3e). These findings suggest that *ChnagG* encodes a 5‐salicylic acid hydroxylase responsible for the conversion of SA to GA.

### 2.4 The Effect of *ChnagG* on Pathogenicity

To further elucidate the function of *ChnagG*, knockout mutants and gene complementation strains of this gene were generated (Figure 4a, Figure S3a,b). Notable differences were observed in the colouration of the colonies on solid medium between the wild type (WT) and the mutants (Figure 4a). The melanin content of the fungi was assessed, revealing that the Δ*ChnagG* mutant produced significantly less melanin than the WT, while the melanin synthesis capability in the complementation strain was restored (Figure 4b). This indicates that the deletion of the *ChnagG* gene adversely affects melanin synthesis. Melanin serves as a virulence factor in *C*. *heterostrophus*, facilitating host invasion. However, the knockout of *ChnagG* did not significantly impact the growth and sporulation of *C*. *heterostrophus* (Figure S3c,d). After 12 h of incubation on cellophane, it was found that the deletion of *ChnagG* had no significant effect on the formation of infection structure appressoria (Figure 4c, Figure S3e), but leaves inoculated with the Δ*ChnagG* exhibited reduced lesion area compared to those inoculated with WT, and the pathogenic ability of the complemented strain C‐Δ*ChnagG* was significantly restored in comparison to Δ*ChnagG* (Figure 4c,d). It was suggested that the deletion of *ChnagG* diminished the pathogenicity.

Because *ChnagG* exhibits SA‐degrading activity, we evaluated the content of SA in maize leaves inoculated with WT, Δ*ChnagG* and C‐Δ*ChnagG* at 72 h post‐inoculation (hpi). The results showed a significant increase in SA content in leaves infected with Δ*ChnagG* compared to those infected with WT (Figure 4e). Isochorismate synthase 1 (*ICS1*) is the key enzyme in SA biosynthesis (Djamei et al. 2011). *NPR1* serves as the receptor of SA, and previous studies reported that maize *ZmNPR1* and *ZmPR1* are upregulated in response to SA and participate in disease resistance in maize (Hernández‐Coronado et al. 2022; Li et al. 2024; Kong et al. 2023). So, we analysed the expression levels of *ZmICS1*, *ZmNPR1* and *ZmPR1*. Compared to uninoculated controls, leaves inoculated with WT showed significant downregulation of *ZmICS1* expression but upregulation of *ZmNPR1* and *ZmPR1* at 72 hpi. In contrast, Δ*ChnagG*‐inoculated leaves exhibited upregulated *ZmICS1* expression relative to uninoculated controls, with higher relative expression levels of *ZmNPR1* and *ZmPR1* compared to WT inoculation (Figure 4f–h). These results indicate that *C*. *heterostrophus ChnagG* facilitates pathogenicity by interfering with SA‐mediated immune responses in maize.

To further elucidate the role of *ChnagG* in SA‐mediated immunity, maize leaves were pretreated with 0.5 mM SA before inoculation. SA application significantly reduced the lesion area in both WT‐ and Δ*ChnagG*‐inoculated leaves compared to untreated controls (Figure S3f,g), confirming SA's critical role in maize resistance to *C*. *heterostrophus*. Notably, Δ*ChnagG*‐inoculated leaves treated with SA displayed the smallest lesion area among all treatments, accompanied by an upregulated relative expression level of *ZmNPR1* compared to WT (Figure S3h). It further supported that *ChnagG* positively regulates the pathogenicity of *C*. *heterostrophus* by suppressing SA‐mediated host resistance.

## 3 Discussion

SA is well recognised for its crucial role in the plant defence system, where the *NPR1* and *NPR3/NPR4* pathways regulate SA‐induced defence gene expression to enhance plant stress tolerance (Fu et al. 2012; Luo et al. 2024). In plant mutants that hinder SA biosynthesis resulting in non‐accumulation of SA, such as transgenic tobacco plants containing the *nahG* gene and SA‐induction‐defective (*sid*) mutants, the capacity to resist pathogenic invasion is significantly compromised (Friedrich et al. 1995; Nawrath and Métraux 1999; Wildermuth et al. 2001). At the same time, various strategies adopted by plant pathogens undermine SA‐mediated plant defence, which also highlights the key role of SA in the process of plant disease resistance (White 1979; Yalpani et al. 1991; Djavaheri et al. 2019). The diverse strategies employed by plant pathogens highlight the vital importance of disrupting SA‐mediated plant defences during disease progression. Gaining a deeper understanding of the impact of SA on pathogens during interactions can expand our understanding of how plant pathogens cause diseases and develop more effective methods to control these fungal diseases. In this study, we observed that the addition of exogenous SA at concentrations less than 1 mM to the culture medium had no effect on the growth or spore germination of *C*. *heterostrophus*. Notably, 1 mM SA concentration far exceeds the endogenous SA levels detected in maize (Pokotylo et al. 2022; Jia et al. 2023). So, we maintain that SA primarily acts as a signalling molecule to activate downstream immune pathways rather than directly inhibiting pathogens. Through transcriptomic and LC–MS analyses, we identified the crucial gene *ChnagG* involved in the SA metabolism of *C*. *heterostrophus*. The creation of mutants demonstrated that the loss of this gene resulted in decreased pathogenicity. At the same time, the content of melanin in fungi decreased after gene knockout. However, melanin is a key pathogenic factor in the infection of *C*. *heterostrophus*; it was found that the deletion of *ChnagG* had no significant effect on the formation of infection structure appressoria. We therefore propose that the impact of gene knockout on pathogenicity is mediated through its disruption of host SA metabolism. Notably, inoculation with the WT did not induce upregulation of SA content or its biosynthesis gene *ZmICS1*, but it did trigger significant upregulation of SA‐responsive genes *ZmNPR1* and *ZmPR1* at 72 hpi. In contrast, the Δ*ChnagG* mutant infection led to elevated SA accumulation, accompanied by upregulated relative expression levels of *ZmICS1*, *ZmNPR1* and *ZmPR1* compared to WT. To validate the role of SA in maize resistance to *C*. *heterostrophus*, leaves were pretreated with 0.5 mM SA before inoculation. SA application significantly reduced disease lesion area in both WT and Δ*ChnagG* inoculated leaves compared to untreated controls, confirming SA's involvement in host resistance. Consequently, *ChnagG* of *C*. *heterostrophus* modulates its pathogenicity by interfering with the host's SA‐mediated immune response. Given that the protein can catalyse the conversion of SA into GA, we propose that such effects are mediated through SA degradation. However, the impact of the degradation product GA on disease resistance‐related signal transduction in the host remains to be further substantiated.

Microorganisms degrade SA through various metabolic pathways. Current research has confirmed that pathogenic fungi eliminate the inhibitory effects of plant SA through the catechol pathway, especially in extensive studies of *F*. *graminearum*, which have demonstrated that SA is converted to catechol under the action of 1‐salicylic acid hydroxylase, inhibiting the plant's immune signalling pathways (Qi et al. 2012, 2018b, 2019b, 2019a; Rocheleau et al. 2019). However, there are currently no reports of GA pathways for SA metabolism existing in fungi. This study detected the presence of GA in *C*. *heterostrophus* cultured with exogenous SA through LC–MS analysis. Transcriptomic analysis showed significant changes in the expression of genes related to carbon metabolic pathways after SA treatment. We speculate that there may be a GA pathway for SA metabolism in *C*. *heterostrophus*. That is, in the case of SA induction, *C*. *heterostrophus* converts SA to gentioic acid, 3‐maleylpyruvate, maleic acid and d‐malate through 5‐salicylic acid hydroxylase (EC:1.14.13.172), gentisate 1,2‐dioxygenase (EC:1.13.11.4), maleyl pyruvic hydrolase (EC:3.7.1.23) and maleate hydratase (EC:4.2.1.31). Furthermore, SA induced differential expression of genes encoding d‐malate dehydrogenase (EC:1.1.1.83) and pyruvate dehydrogenase E1 (EC:1.2.4.1) and E2 (EC:2.3.1.12), leading us to believe that SA is further metabolised into pyruvate and acetyl‐CoA via GA metabolic products, subsequently entering the TCA cycle (Pietrocola et al. 2015; Shi and Tu 2015). Moreover, *ChnagG* deletion through the creation of mutants impacted the synthesis of fungal melanin. The melanin in *C*. *heterostrophus* is a 1,8‐dihydroxynaphthol polymer that synthetic substrate is acetyl‐CoA (Tanaka et al. 1992; Langfelder et al. 2003). The results showed that there was potential crosstalk between SA metabolism and the melanin biosynthesis pathway in fungi.

The enzyme responsible for catalysing the synthesis of genipin from SA has been identified in bacteria. In *Pseudomonas* sp. strain U2, a gene cluster operates synergistically through multiple genes. The first open reading frame, termed *nagG*, encodes a protein homologous to the large subunit of a dihydroxylating dioxygenase featuring a Rieske‐type iron–sulphur centre. The second open reading frame, designated *nagH*, encodes a protein homologous to the small subunit of the same dioxygenase. Together, *NagG*, *NagH* and *NagAb* facilitate the conversion of SA into GA, thereby exhibiting 5‐salicylic acid hydroxylase activity. However, we identified only one candidate gene (*ChnagG*) for 5‐salicylic acid hydroxylase; the homologous genes of other genes were not successfully aligned compared in *C*. *heterostrophus* (Fuenmayor et al. 1998). This gene encodes a protein that contains the domains Rieske Ro_Alpha_N and Ring hydroxylating α subunit. The Rieske iron–sulphur centre comprises a [2Fe‐2S] cluster that is involved in electron transfer, while Ring_hydroxyl_A serves as the catalytic domain of the aromatic‐ring‐hydroxylating dioxygenase system, featuring an active site with a non‐heme ferrous ion coordinated by three ligands. Both domains are capable of executing functions analogous to those of the NagG protein. Subsequent prokaryotic heterologous expression experiments proved that the gene could exert the role of 5‐salicylic acid hydroxylase on its own, indicating that there were differences in the mechanism of action of enzymes that catalyse SA to GA in bacteria and fungi. Furthermore, additional studies indicated that the metabolic capacity for exogenous SA in the strain remained largely intact following the deletion of the *ChnagG* gene. We assessed the expression level of the homologous gene COCC4DRAFT_124440, which encodes salicylic acid hydroxylase (E1.14.13.1) from *F*. *graminearum* in *C*. *heterostrophus* (Qi et al. 2019b); notably, the expression level of COCC4DRAFT_124440 was significantly upregulated in the absence of the *ChnagG* gene compared to WT (results not shown). Thus, it is hypothesised that this gene may offset the metabolic consequences arising from the deletion of the *ChnagG* gene on SA metabolism. This suggests the existence of multiple metabolic pathways for SA in *C*. *heterostrophus*, underscoring the need for further investigation to elucidate the molecular mechanisms governing the genipin metabolic pathway during the infection process of *C*. *heterostrophus*.

## 4 Experimental Procedures

### 4.1 Fungal and Plant Culture Conditions

The materials for the fungus (*C. heterostrophus*) were found in nature as one of two races, T and O. The culture of *C*. *heterostrophus*, the cultivation of conidia, and the infection of plants by the pathogen were conducted in accordance with the experimental procedures outlined previously (Wu et al. 2012). The SA used in the fungal hyphal growth test was sourced from a 1 or 10 mM methanol stock solution, with 1 mL of methanol included in the control treatment without SA. Mycelial growth was assessed by measuring the diameter of a single colony over 7 consecutive days. The experimental conditions for the medium pH effect on fungal hyphal growth included pH levels of 4, 5, 6, 7 and 8. Following incubation on SA‐containing water agar medium at 25°C for approximately 6 h, the germination rate of conidia was assessed, and the number of germinated conidia was recorded.

The plant variety used in this study was B73. The exogenous SA test in plants, 0.5 mM SA was sprayed on the back of maize leaves and then inoculated with pathogenic fungi. Maize leaves of different stages were collected. The SA content of leaves was measured by weighing a sample of 1.0 ± 0.1 g, grinding it, and dissolving it in phosphate‐buffered saline (PBS, pH 7.4) solution at a wt/vol ratio of 1:9. The subsequent steps were carried out according to the instructions of the plant SA content ELISA kit (Wuhan Mosak Biotechnology). The absorbance value of the sample was measured at 450 nm, and the SA content in the sample was determined using the standard curve.

### 4.2 Transcriptome Sample Collection, Extraction and Measurement

Mycelial samples of *C*. *heterostrophus* were cultivated in liquid medium for 3 days. Following the filtration of the medium, the samples were rapidly frozen in liquid nitrogen and stored at −80°C to preserve RNA quality prior to sequencing the library of *C*. *heterostrophus* using the Illumina NovaSeq 6000 platform. Paired‐end clean reads were mapped to the reference genome (SRX25383940, ID:33841682) to establish an index of the reference genome using HISAT2 v. 2.4. All DEGs were mapped to Gene Ontology (GO) terms in the ontology database (with calculations for each term available at http://www.geneontology.org/), and KEGG analysis was conducted in accordance with the procedures outlined in Reactome. The original sequencing data were processed using FastQC for quality control, and subsequent analyses used the HISAT2 aligner to map reads to the reference genome (GCF 000354255.1). Differential gene expression analysis was performed using the DESeq2 package in R, with a false discovery rate (FDR) threshold of < 0.05 considered statistically significant. Functional annotation and pathway analysis were conducted using the DAVID and KEGG databases.

### 4.3 RT‐qPCR Measurement of Relative Expression Levels of Target Genes

All RNA samples were rapidly frozen in liquid nitrogen immediately after collection and subsequently stored at −80°C. Total RNA was extracted using a fungal RNA column extraction kit (Sangon). Complementary DNA (cDNA) was synthesised using a one‐step RT‐PCR amplification kit (TransGen Biotech), and quantitative real‐time PCRs were conducted using the manufacturer's two‐step system (TransGen Biotec), with a reaction volume of 10 μL. *Actin* served as the internal control for RT‐qPCR analysis. Specific primers for the candidate genes of *C*. *heterostrophus* were designed using Primer3.0 software based on the protein sequences of target genes from the KEGG database, and the specificity of the primers was verified using NCBI resources. All primers were synthesised by Sangon. Experimental data were analysed using the 2^−ΔΔ*C*t^ method to determine relative gene expression levels. The RT‐qPCR primers are presented in Tables [Link], [Link] and [Link], [Link], and the expression levels of each gene were calculated using three biological replicates and three technical replicates.

### 4.4 Metabolites Identification

For the HPLC detection of SA content in liquid medium, the culture medium of *C*. *heterostrophus*, which had been grown in SA for 3 days, was extracted using ethyl acetate. Subsequently, the extract was purged with nitrogen to replace the solvent with acetonitrile. The mobile phase consisted of acetonitrile (90%) as phase A and 0.1% formic acid in water (10%) as phase B, with a flow rate of 0.3 mL/min. The column temperature was maintained at 40°C, and the metabolites were separated and detected using a Shimadzu LC‐20A liquid chromatography system equipped with a C18 chromatographic column.

For LC–MS detection of fungal metabolites, samples of *C*. *heterostrophus* cultured for 3 days in a medium with 0.5 mM SA were selected alongside the transcriptome. After grinding the sample, the supernatant was collected, and methanol was added to achieve a final concentration of 53%. Metabolites were screened through a C18 column at a flow rate of 0.2 mL/min at 40°C. In positive ion detection mode, the mobile phase consisted of 0.1% formic acid and methanol, while in negative ion mode, mobile phase A was adjusted to 5 mM ammonium acetate (pH 9.0). The scanning range was set to *m*/*z* 100–1500. The settings for the ESI source were as follows: spray voltage: 3.2 kV; sheath gas flow rate: 40 arb; auxiliary gas flow rate: 10 arb; capillary temperature: 32°C. Both positive and negative polarities were employed, with MS/MS level 2 scans being data‐dependent. The data were compared with mzCloud (https://www.mzcloud.org/) and mzVault against the Masslist database. Blank samples were used to eliminate background ions, and the quantitative results were normalised, ultimately leading to the identification and quantification of the metabolites.

The purified protein was subjected to enzyme activity assays and LC–MS detection, conducted in strict accordance with the method of Qi et al. (2019b). For the detection of fungal metabolites, mycelia cultivated for 3 days in complete medium (CM) supplemented with 50 mM SA were collected, ground, and dissolved in PBS, from which the metabolites were extracted. The detection of SA content in plants was carried out using an ELISA kit (MSKBIO).

### 4.5 Bioinformatics Analysis of Target Genes

The sequences of the target genes were obtained from NCBI, and protein sequence alignments along with heatmap generation were performed using TBtools. A phylogenetic tree was constructed using MEGA X, while protein domain information was predicted using InterPro (https://www.ebi.ac.uk/interpro/search/sequence/).

### 4.6 Construction of the pET32a‐ *ChnagG* Prokaryotic Expression Vector

The gene fragment of *ChnagG* was amplified using cDNA from *C*. *heterostrophus* as a template and subsequently ligated with pET32a via the seamless cloning method at the BamHI and EcoRI restriction sites. The resulting construct was transformed into *Escherichia coli* strains DH5α and BL21, and the correctness of the sequence was confirmed through sequencing. Following the screening of positive clones for optimal induction conditions, the recombinant protein was purified using the His protein purification kit from Sangon and verified by SDS‐PAGE. Enzyme activity assays and LC–MS detection were then performed according to Qi's methods. Tables [Link], [Link] and [Link], [Link] lists the primers used in the experimental assays.

### 4.7 Creation and Analysis of Knockout Mutants

To construct the *ChnagG* knockout mutant, overlapping PCR was used to amplify 1 kb upstream and downstream fragments of the target gene coding region, and the upstream and downstream halves were fused with the hygromycin (*HPH*) gene. For gene deletion transformation, a polyethylene glycol (PEG)‐mediated protoplast transformation method was used to transform at least 5 μg of DNA into race T. The transformants were screened three times on potato dextrose agar (PDA; BioFroxx) containing hygromycin, and the DNA of the transformants was extracted for preliminary screening of knockout mutants. Single spores of the cultured mutants were then screened and identified as gene knockout mutants verified by DNA and RT‐qPCR (Zainudin et al. 2015). The method for extracting melanin strictly followed the protocol (Zhang et al. 2021). Tables [Link], [Link] and [Link], [Link] lists the primers used in the experimental assays.

## Conflicts of Interest

The authors declare no conflicts of interest.

## Supporting information


**Figure S1.** Growth of the corn leaf spot pathogen in different pH media. (a) Phenotypes of *Cochliobolus heterostrophus* cultivated for 5 days on complete medium (CM) across pHs of 4, 5, 6, 7 and 8. (b) Growth diameter of race T on CM at varying pHs. The diameters of the fungal colonies were measured at 3, 4 and 5 days, with significant difference analysis and growth inhibition rate analysis conducted on the data collected from day 5. The data are presented as the mean ± SD based on triplicate measurements from a representative experiment. Significant differences between groups (*p* < 0.05) were analysed by one‐way ANOVA, groups labelled with the same lowercase letter (a, b and c) are not statistically different.

**Figure S2.** GO analysis, featuring a combined analysis of group data for comparisons between E1 versus CK and E2 versus CK. The GO terms included in the database, arranged from top to bottom, are molecular function (MF), cellular component (CC) and biological process (BP). Red indicates upregulated differential genes, while blue signifies downregulated differential genes.

**Figure S3.** Verification of fungal mutant creation and measurement of salicylic acid (SA) levels in leaves. (a) DNA validation of the *ChnagG* gene in knockout and complementation strain transformants. (b) Reveres transcription‐quantitative PCR validation of the *ChnagG* gene in various strains assessed via agarose gel. The data are presented as the mean ± SD based on triplicate measurements from a representative experiment. Significant differences between groups (*p* < 0.05) were analysed by one‐way ANOVA, groups labelled with the same lowercase letter (a, b and c) are not statistically different. Similar results were observed in follow‐up independent experiments. (c) Statistics of colony growth diameter (day 3 of growth in complete medium) of different strains. (d) Statistics on the number of conidia produced by different strains. (e) Statistics on the number of appressorium (AP) (incubated on cellophane for 12 h) of different strains. (f, g) Maize leaves inoculated with wild type (WT_, Δ*ChnagG* and C‐Δ*ChnagG*) treated with SA (0.5 mM) and water as control. (h) Relative expression levels of *ZmNPR1* in 72 h post‐inoculation of maize leaves inoculated with water, WT, Δ*ChnagG* and C‐Δ*ChnagG*.

**Table S1** Identification of metabolites via liquid chromatography‐mass spectrometry (LC–MS).

**Table S2** Primers used in this study.

## Data Availability

The data that support the findings of this study are available from the corresponding author upon reasonable request.
