# Supplementary figures and images for: ChnagG Plays the Role of 5‐Salicylate Hydroxylase in the Gentisic Acid Pathway of Salicylic Acid Metabolism in Cochliobolus heterostrophus
Source: Mol Plant Pathol. 2025 Jun 2;26(6):e70090. doi: 10.1111/mpp.70090 (PMC12130555; doi:10.1111/mpp.70090)

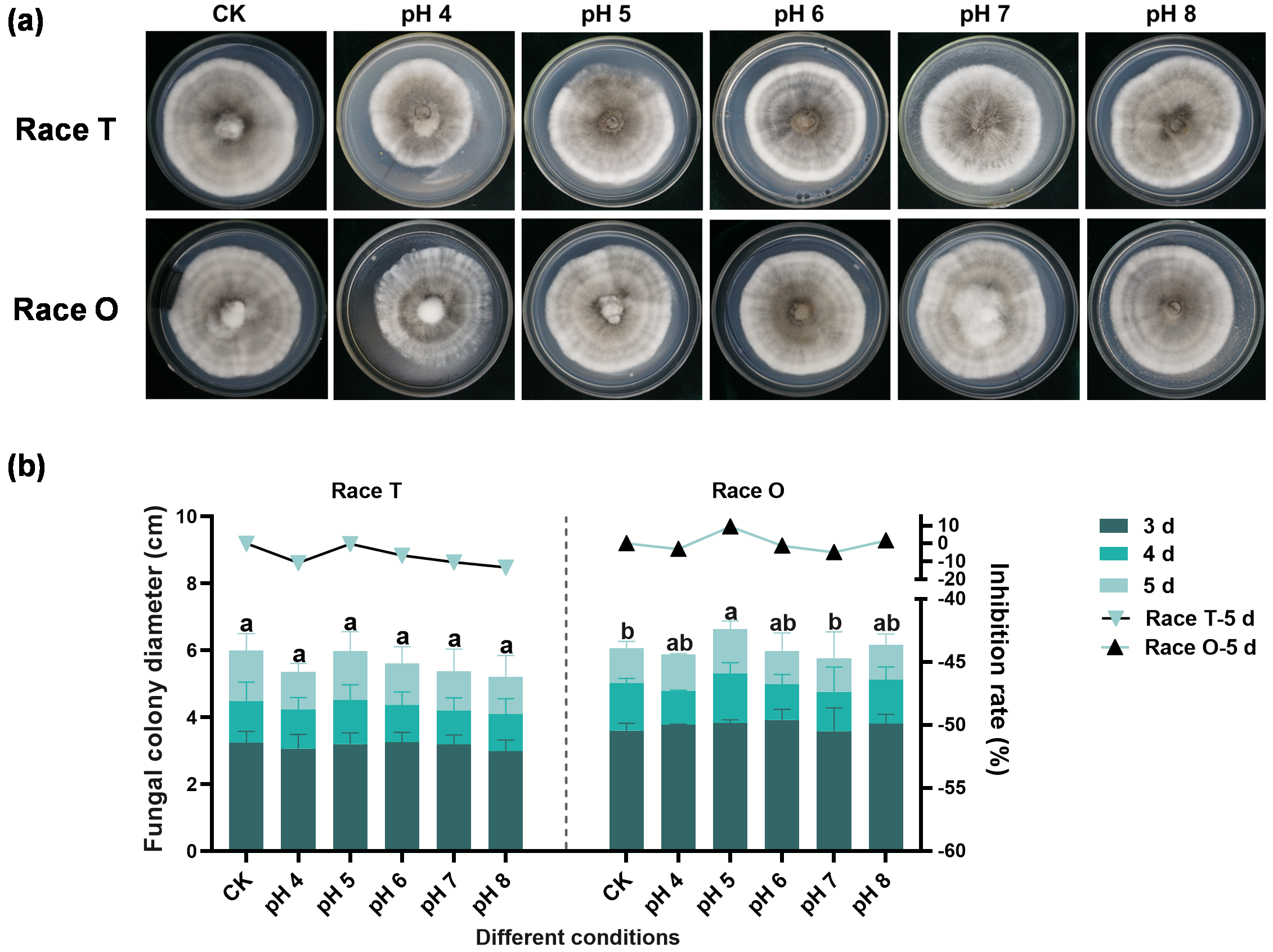

Supplement: Supplementary file 1 — Figure S1. Growth of the corn leaf spot pathogen in different pH media. (a) Phenotypes of Cochliobolus heterostrophus cultivated for 5 days on complete medium (CM) across pHs of 4, 5, 6, 7 and 8. (b) Growth diameter of race T on CM at varying pHs. The diameters of the fungal colonies were measured at 3, 4 and 5 days, with significant difference analysis and growth inhibition rate analysis conducted on the data collected from day 5. The data are presented as the mean ± SD based on triplicate measurements from a representative experiment. Significant differences between groups (p < 0.05) were analysed by one‐way ANOVA, groups labelled with the same lowercase letter (a, b and c) are not statistically different. [file MPP-26-e70090-s001.tif]

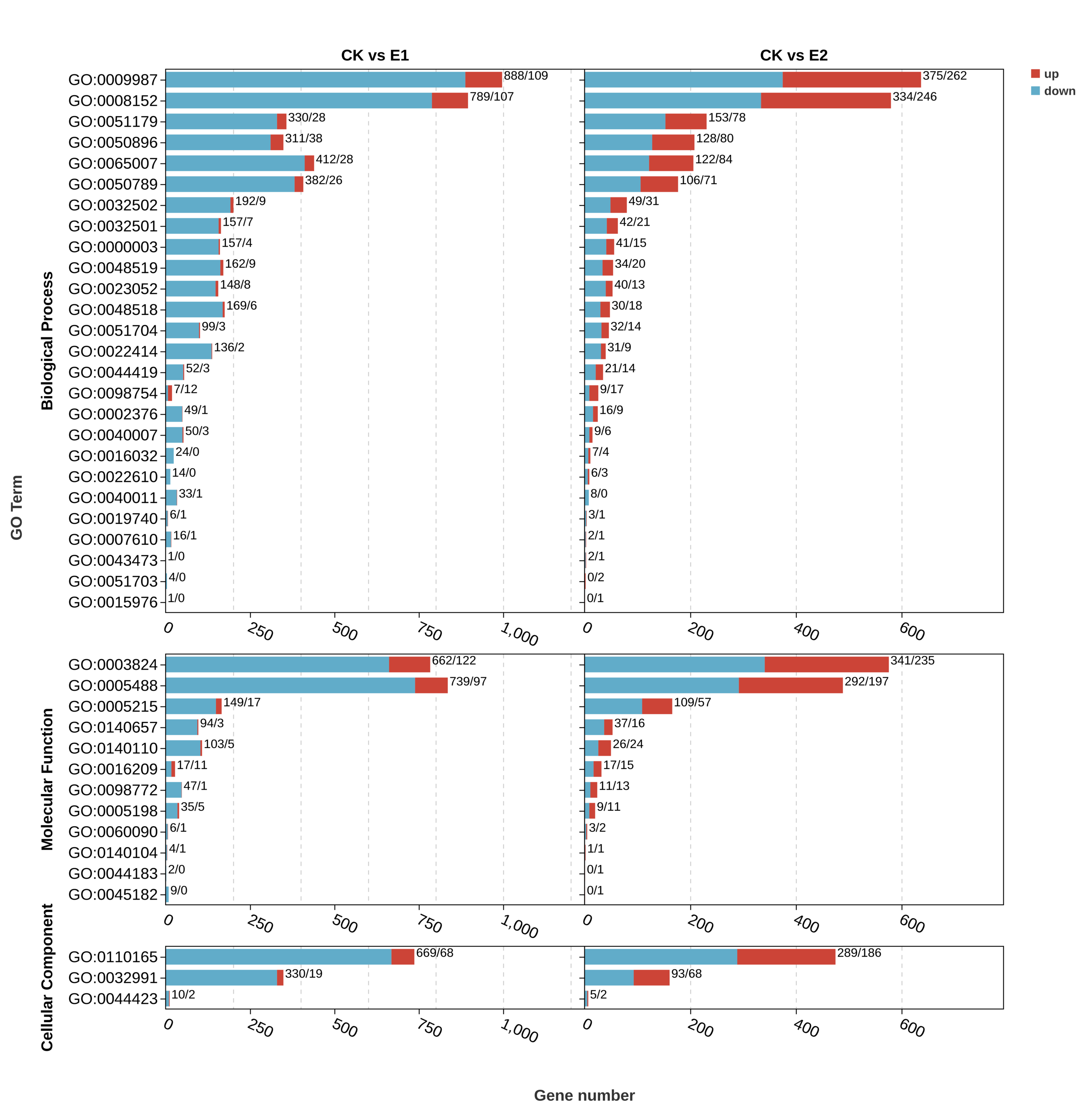

Supplement: Supplementary file 2 — Figure S2. GO analysis, featuring a combined analysis of group data for comparisons between E1 versus CK and E2 versus CK. The GO terms included in the database, arranged from top to bottom, are molecular function (MF), cellular component (CC) and biological process (BP). Red indicates upregulated differential genes, while blue signifies downregulated differential genes. [file MPP-26-e70090-s002.tiff]

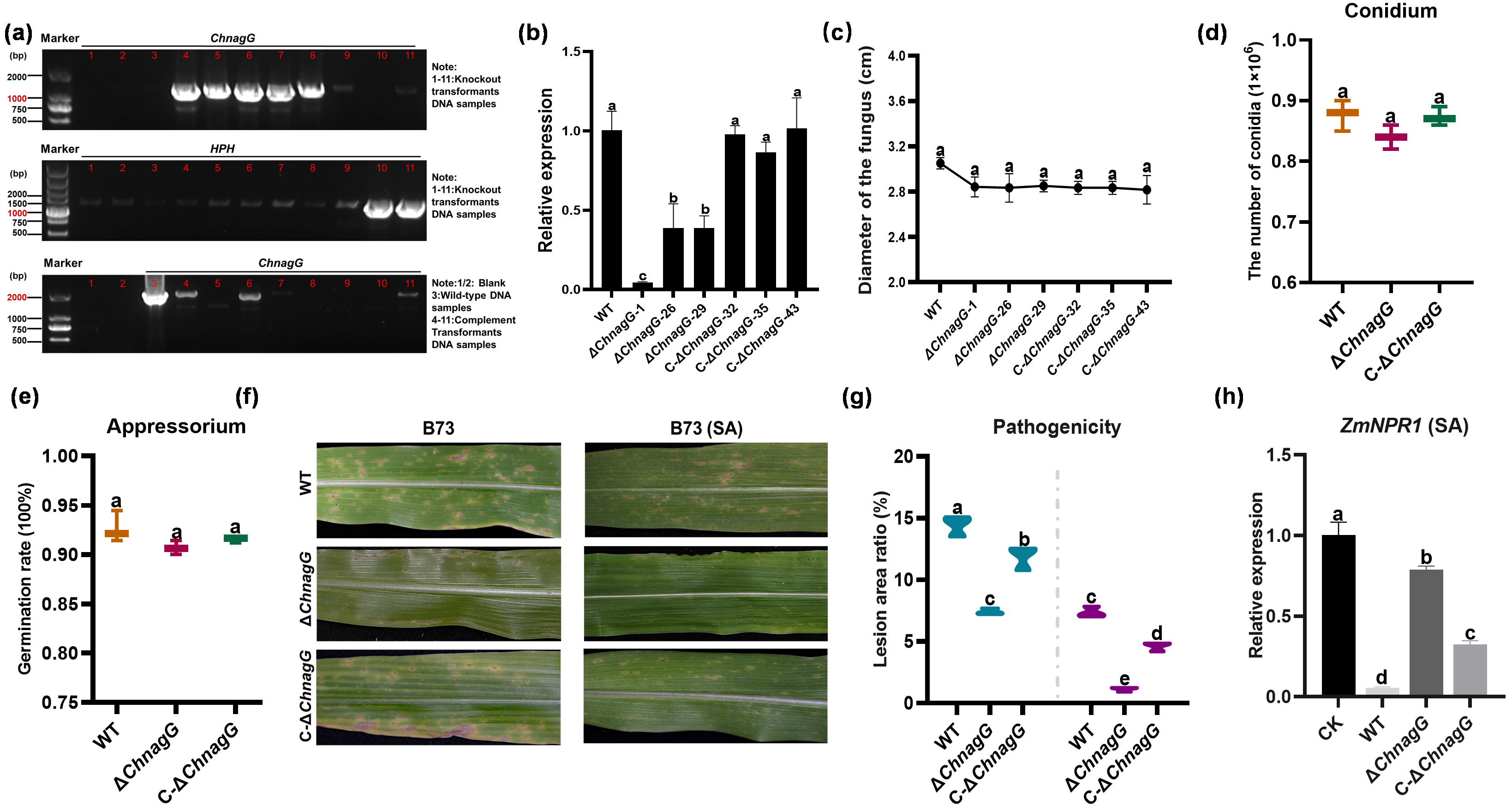

Supplement: Supplementary file 3 — Figure S3. Verification of fungal mutant creation and measurement of salicylic acid (SA) levels in leaves. (a) DNA validation of the ChnagG gene in knockout and complementation strain transformants. (b) Reveres transcription‐quantitative PCR validation of the ChnagG gene in various strains assessed via agarose gel. The data are presented as the mean ± SD based on triplicate measurements from a representative experiment. Significant differences between groups (p < 0.05) were analysed by one‐way ANOVA, groups labelled with the same lowercase letter (a, b and c) are not statistically different. Similar results were observed in follow‐up independent experiments. (c) Statistics of colony growth diameter (day 3 of growth in complete medium) of different strains. (d) Statistics on the number of conidia produced by different strains. (e) Statistics on the number of appressorium (AP) (incubated on cellophane for 12 h) of different strains. (f, g) Maize leaves inoculated with wild type (WT_, ΔChnagG and C‐ΔChnagG) treated with SA (0.5 mM) and water as control. (h) Relative expression levels of ZmNPR1 in 72 h post‐inoculation of maize leaves inoculated with water, WT, ΔChnagG and C‐ΔChnagG. [file MPP-26-e70090-s003.tif]
